# Supplementary material for: The Influence of Sociodemographic Factors on Symptoms of Anxiety, the Level of Aggression and Alcohol Consumption in the Time of the COVID-19 Pandemic among Polish Respondents
Source: Int J Environ Res Public Health. 2022 Jun 9;19(12):7081. doi: 10.3390/ijerph19127081 (PMC9222902; doi:10.3390/ijerph19127081)
Supplement: Supplementary file 1 [file ijerph-19-07081-s001.zip › ijerph-1699704-supplementary.pdf]

Table S1. Structure of the studied group in comparison to data from the Statistical Yearbook of the Central Statistical Office of Poland 2019.

	Number of respondents	Percentage value to the number of respondents [%]	Percentage values to the Polish population according to GUS [%]
Number of respondents	538	100	-
Sex:			
• men	125	23.23	47.73
• women	413	76.77	52.27
Age:			
• 18-29	366	68.03	Working-age population: **60.58**
• 30-49	147	27.32	
• 50+	25	4.65	
Domicile:			
• village	105	19.52	39.90
• city	433	80.48	60.10
Education:			
• basic	7	1.30	19.2
• professional	1	0.19	20.8
• medium	254	47.21	31.9
• higher	271	50.37	26.4
• no answer	5	0.93	-
